# Recent Advances in the Biological Activity of s-Triazine Core Compounds

**DOI:** 10.3390/ph15020221

**Published:** 2022-02-12

**Authors:** Dawid Maliszewski, Danuta Drozdowska

**Affiliations:** Department of Organic Chemistry, Medical University of Bialystok, 15-222 Białystok, Poland

**Keywords:** 1,3,5-triazine, s-triazine, anticancer, enzyme inhibitory activity

## Abstract

An effective strategy for successful chemotherapy relies on creating compounds with high selectivity against cancer cells compared to normal cells and relatively low cytotoxicity. One such approach is the discovery of critical points in cancer cells, i.e., where specific enzymes that are potential therapeutic targets are generated. Triazine is a six-membered heterocyclic ring compound with three nitrogen replacing carbon-hydrogen units in the benzene ring structure. The subject of this review is the symmetrical 1,3,5-triazine, known as s-triazine. 1,3,5-triazine is one of the oldest heterocyclic compounds available. Because of its low cost and high availability, it has attracted researcher attention for novel synthesis. s-Triazine has a weak base, it has much weaker resonance energy than benzene, therefore, nucleophilic substitution is preferred to electrophilic substitution. Heterocyclic bearing a symmetrical s-triazine core represents an interesting class of compounds possessing a wide spectrum of biological properties such as anti-cancer, antiviral, fungicidal, insecticidal, bactericidal, herbicidal and antimicrobial, antimalarial agents. They also have applications as dyes, lubricants, and analytical reagents. Hence, the group of 1,3,5-triazine derivatives has developed over the years. Triazine is not only the core amongst them, but is also a factor increasing the kinetic potential of the entire derivatives. Modifying the structure and introducing new substituents makes it possible to obtain compounds with broad inhibitory activity on processes such as proliferation. In some cases, s-triazine derivatives induce cell apoptosis. In this review we will present currently investigated 1,3,5-triazine derivatives with anti-cancer activities, with particular emphasis on their inhibition of enzymes involved in the process of tumorigenesis.

## 1. Introduction

As far as we know, tumors are the most serious cause of death in the world. Cancers with the highest mortality rates in 2018 were lung cancer (2.1 million new cases and 1.8 million deaths), breast cancer (million new cases and 880 thousand deaths), prostate cancer (1.3 million new cases and 360 thousand deaths), and stomach cancer (1 million new cases and 783 thousand deaths) [1].

The fight against cancer has consumed huge amounts of money to find the cure with little effect. Nevertheless, it cannot be defined as a failure. As Napoleon Hill said, “every adversity, every failure, every heartache carries with it the seed of an equal or greater benefit”. Following this thought, we would like to highlight two aspects of the fight against cancer. First, decades of research lead to more and more precise descriptions of the mechanisms taking place in cancer cells, it is possible to determine the most effective aim in targeted therapies. Second and equally important, the development of small molecules. The development of more active, selective and less cytotoxic drugs is due to designing chemical compounds based on a structure-activity relationship (SAR) [2]. In this search, the leading linker is 1,3,5-triazine, a symmetrical heterocyclic aromatic ring enabling the expansion of the structure in a multi-vector manner. Decades of research have revealed a wide range of properties of s-triazine derivatives. In this review we will present currently investigated 1,3,5-triazine derivatives with anti-cancer activities.

This review presents the current state of knowledge on 1,3,5-triazine derivatives, their structures and anticancer activity, as well as their ability to inhibit different enzymes or their DNA-binding potential. This data could be helpful in the development of new drugs and therapeutic methods. By analysing the presented approach, a series of compounds with high potency and low toxicity can be designed, synthesized, characterized and evaluated for desired pharmacological activity. The collected data are presented in summary Table 1.

## 2. Results

### 2.1. Topoisomerase Inhibitors

Topoisomerases are a group of enzymes involved in replication, they are responsible for the degree of twist of the double helix. Topoisomerases convert the chemical energy from ATP into the energy of the torsion tension of a molecule with a superhelical structure. In vivo, topoisomerases unravel the DNA double helix, thus providing a template for the replication or transcription of enzymes. Depending on the number of phosphodiester bonds to be broken at one time, there are two types of enzyme. Topoisomerase I hydrolyses one bond, cuts one strand and is responsible for removing superstrands from the DNA molecule (relaxation). Topoisomerase II hydrolyses two bonds, cuts both strands and is responsible for adding supercoils to the DNA molecule [39].

**Figure 1:**
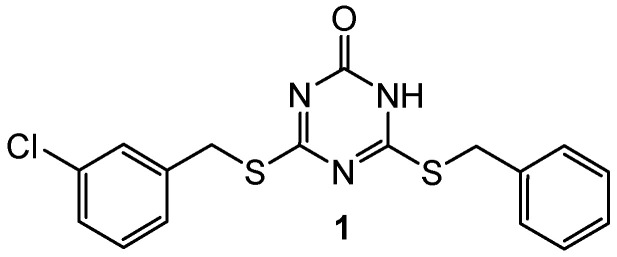
Structure of topoisomerase II inhibitor.

Human topoisomerase II inhibitory properties were shown by 4-(benzylthio)-6-((3-chlorobenzyl)thio)-1,3,5-triazin-2(1H)-one **1** (Figure 1), giving an IC50 of 57.6 µM. Additionally, the binding of compound **1** with the htIIα ATPase domain was proved via microscale thermophoresis (MST) and molecular dynamics (MD) [3].

### 2.2. Dual Phosphoinositide 3-Kinase and Mammalian Target of Rapamycin Inhibitors

The phosphoinositide 3-kinase (PI3K) enzymes show a two-way activity including the activity of the lipid kinase and the activity of the protein kinase. They play a crucial role in processes such as proliferation, migration, differentiation, survival, and trafficking. The PI3K family contains eight isoforms divided into three distinct classes (I, II, and III) which may be different in terms of cellular responsibility [40]. 

The function of the mammalian target of rapamycin (mTor) is to regulate growth, proliferation and cell traffic, and the processes of translation and transcription. The mTOR catalyzes the phosphorylation ribosomal protein S6 kinase β-1 (S6K1), eukaryotic translation initiation factor 4E binding protein **1** (4E-BP1), Akt, protein kinase C (PKC), and type-I insulin-like growth factor receptor (IGF-IR), thereby regulating protein synthesis, nutrient metabolism, growth factor signaling, cell growth, and migration [41]. 

The construction of compounds with dual inhibitory effects contributes to obtaining a more selective effect. Potential anti-cancer drugs that inhibit PI3K and mTor at the same time showed greater efficiency and reduced the likelihood of inducing drug resistance [42]. 

**Figure 2:**
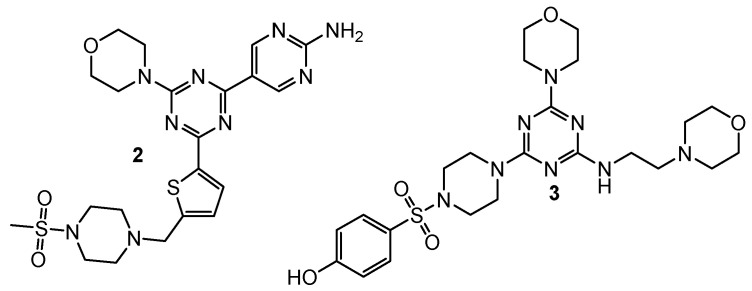
Structure of dual PI3K and mTor inhibitors.

Substituted 2-(thiophen-2-yl)-1,3,5-triazine derivative **2** (Figure 2) exhibited excellent anti-cancer potency for A549, MCF-7 (breast cancer) and Hela (cervical cancer) cell lines with IC50 values of 0.20 μM, 1.25 μM, and 1.03 μM, respectively. Western blot analysis proved drivative **2** could suppress the phosphorylation of AKT. The degree of inhibition (%) demonstrated selective inhibition of PI3Kα/mTOR, unlike epidermal growth factor receptors (EGFR, c-Met, VEGFR-2, and EGFRL858R/T790M) [4].

From the new series of 1,3,5-triazine derivatives rich in morpholine moiety, 4-((4-(4-morpholino-6-((2-morpholinoethyl)amino)-1,3,5-triazin-2-yl)piperazin-1-yl)sulfonyl)phenol **3** (Figure 2) showed the highest cytotoxic activity against MDA-MB321 (breast cancer), MCF-7, HeLa, and HepG2 (human hepatocellular carcinoma) cells with IC50 values of 15.83 μM, 16.32 μM, 2.21 μM, and 12.21 μM, respectively. Kinase inhibitory activity (IC_50_) of derivative **3** was equal to 3.41 nM for PI3K, and 8.45 nM for mTor [5].

### 2.3. Dihydrofolate Reductase Inhibitors

Dihydrofolate reductase (DHFR) is an enzyme responsible for reducing dihydrofolic acid to tetrahydrofolic acid by catalyzing the transfer of hydride from NADPH, generating the oxidized form of NADP^+^ [43]. Inhibiting DHRF induces an amount reduction of tetrahydrofolate (THF), consequently decreasing the synthesis of purines, amino acids, and thymidylate, which are crucial in cell growth and proliferation [44].

**Figure 3:**
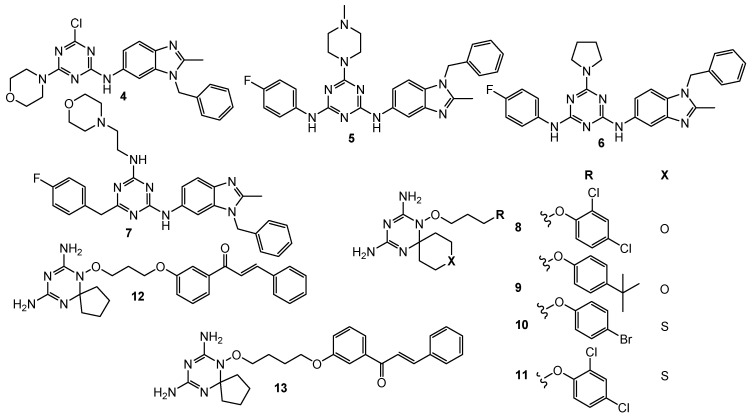
Structure of DHFR inhibitors.

Singa et al. demonstrated synthesized triazine-benzimidazole analogs **4**–**7** (Figure 3) appointed with a hydrogen bond interaction domain, a polar hydrophilic substituent and an intercalating group. The median growth inhibitory (GI_50_) values for these compounds were measured relative to leukemia, non-small cell lung cancer, colon cancer, central nervous system (CNS) tumor, melanoma, ovarian cancer, renal cancer, prostate cancer, and breast cancer cells with values in the range of 1.91–2.72 µM. The 50% inhibitory concentration value of DHRF activity was lowest for derivative **7** and was 0.002 µM, which was equivalent to methotrexate (MTX) (IC50 = 0.02 µM) [6,45].

Zhou et al. reported hDHFR inhibiting activity in four 1,3,5-triazine analogs bearing a heteroatom (O/S) spiro-ring. Structures **8**–**11** (Figure 3) presented hDHFR inhibitory activity with IC_50_ values of 7.46 nM, 3.72 nM, 6.46 nM, and 4.08 nM, compared with MTX. An in vivo study demonstrated that compound **8** significantly inhibited tumor growth in a nude mouse [7]. 

A hybrid of 4,6-diamino-1,2-dihydro-1,3,5-triazine and chalcone led to the generation of 15 new compounds as potential DHFR and TrxR (thioredoxin reductase) inhibitors. The greatest results were exhibited by compounds **12** and **13** (Figure 3). Both acted cytotoxic against HCT116 (human colorectal carcinoma) (GI_50_ = 0.026 µM; GI_50_ = 0.116 µM) and MCF-7 (GI_50_ = 0.080 µM; GI_50_ = 0.127 µM) cancer cell lines. In addition, studies have shown strong in vitro inhibitory activities against recombinant human DHFR (IC_50_ = 0.0061 µM; IC_50_ = 0.0026 µM) and rat TrxR (IC_50_ = 4.6 µM; IC_50_ = 5.9 µM) enzymes [8].

### 2.4. Carbonic Anhydrase Inhibitors

Carbonic anhydrases (CAs), metalloenzymes from the lyase group, are responsible for pH homeostasis and catalyzing the reversible reaction of the formation of the bicarbonate ion ${\mathrm{HCO}}_{3}^{-}$ from water and carbon dioxide [46].

**Figure 4:**
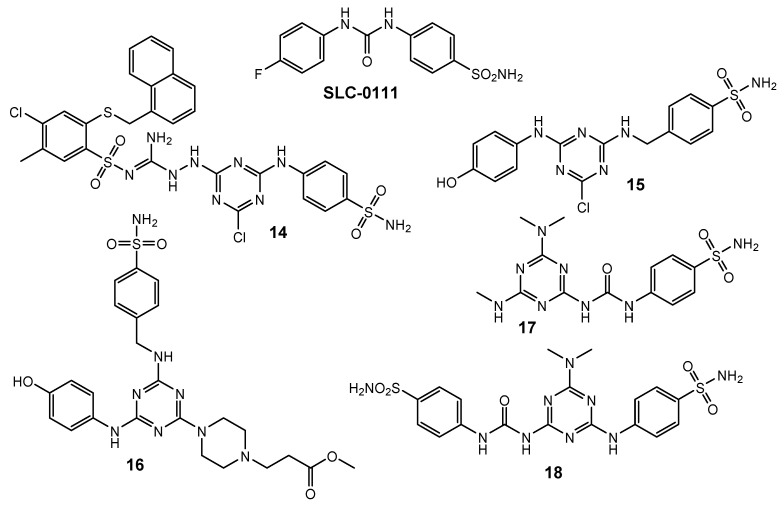
Structure of CA inhibitors.

Among the numerous isoforms we can distinguish the ubiquitous variants CA I and CA II in mammals. In a pathological condition such as hypoxia, increased expression of CA IX and CA XII is observed. These enzyme forms are involved in the regulation of pH homeostasis and intercellular communication and ion transport. 2-[4-Chloro-5-methyl-2-(naphthalen-1-ylmethylthio)-benzenesulfonyl]-1-[4-chloro-6-(4-sulfamoylphenylamino)-1,3,5-triazin-2-ylamino]guanidine **14** (Figure 4) acted with strongest selectivity toward hCA IX versus hCA I (hCA I/hCA IX = 18) and hCA II (hCA II/hCA IX = 4). Compound **14** showed prominent cytotoxicity towards HeLa cancer cells (IC_50_ = 17 µM) and did not exhibit toxicity to the non-cancerous HaCaT cells (IC_50_ = 61 µM) [9].

Research conducted by Havránková et al. considered the interaction of CA I, II and IX with 1,3,5-triazine derivatives incorporating piperazine, aminoalcohol and sulfonamide. The results showed that 1,3,5-triazines with a 4-hydroxyaniline substituent achieved the highest ratio of selective inhibition (hCA IX/hCA II): compound **15** (18.50); compound **16** (14.09) (Figure 4) [10].

Based on the structure of SCL-0111, new 1,3,5-triazine derivatives **17** and **18** were synthesized (Figure 4) and their ability to inhibit CA I, II, IX, and XII was investigated. The most promising result was the selective inhibition of CA IX by compound **17** with a KI value = 0.91 nM [11], while compound **18** had a KI value of 14.6 nM [12].

### 2.5. Epidermal Growth Factor Receptor Inhibitors

The role of the epidermal growth factor receptor (EGFR) in the pathogenesis process is an important topic of scientific research. As a result, it was discovered that mutations leading to overexpression of EGFR genes (e.g., increased regulation or amplification) are significantly associated with many cancers: lung granuloma (40% of cases), rectal tumors, glioblastoma (50%), and epithelial carcinomas of the head and neck (80–100%) [47,48]. 

**Figure 5:**
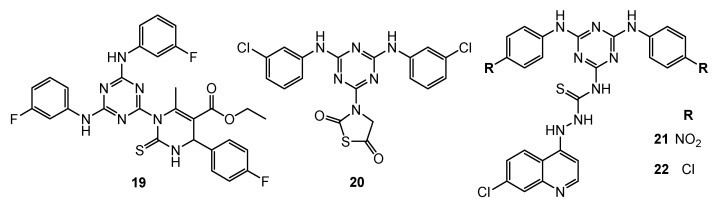
Structure of EGFR inhibitors.

Through the “one pot” reaction, 15 novel monastrol-1,3,5-triazine derivatives were obtained and investigated for anti-cancer properties and cytotoxicity. Derivative **19** substituted by 3-fluorphenylamino groups (Figure 5) presented highest IC50 against cancer cell lines [HeLa—39.7 µM; MCF-7—41.5 µM; HL-60 (human pro-myelocytic leukemia cell)—23.1 µM; HepG2—31.2]. This compound was nontoxic to normal epithelial cells MCF-12A while at a concentration of 10 nM the inhibition of EGFR-TK by **19** was equal 96.4% [13].

Analysis of molecular modelling and Lipinski’s rule of five allowed us to select four compounds that were tested for anti-breast cancer activity. The strongest action with respect to EGFR-TK was observed for 3-(4,6-bis((3-chlorophenyl)amino)-1,3,5-triazin-2-yl)thiazolidine-2,5-dione **20** (Figure 5) (IC_50_ = 2.54 µM). An in vitro study against MDA-MB-21, BT-474 (breast tumor) and MCF-7 showed an increase of apoptosis rates. In addition, a significant decline expression of β-catenin was noticed in MDA-MB-21 cell lines [14]. 

Bhat et al. took a closer look at 4-aminoquinoline-1,3,5-triazine derivatives. Compounds **21** (Figure 5) presented IC_50_ values of 44.5 µM, 52.2 µM, 40.3 µM, and 56.4 µM against HeLa, MCF-7, HL-60, and HepG2. Derivative **22** (Figure 5) showed IC_50_ values of 32.4 µM, 32.3 µM, 26.3 µM, and 45.3 µM against HeLa, MCF-7, HL-60, and HepG2. Both molecules did not reveal cytotoxicity to MCF-12A cells. The activity of derivatives **21** and **22** inhibiting EGFR-TK was 96.3% and 90.5%, respectively [15].

### 2.6. Vascular Endothelial Growth Factor

Vascular endothelial growth factor (VEGF) production can be induced in a cell that does not receive enough oxygen [49]. When a cell is deficient in oxygen, it produces hypoxia induced factor (HIF). HIF stimulates the release of VEGF (including the modulation of erythropoiesis). Circulating VEGF then binds to VEGF receptors on endothelial cells, triggering a tyrosine kinase pathway leading to angiogenesis [50]. Expression of angiopoietin-2 in the absence of VEGF leads to endothelial cell death and vascular regression. VEGF acts as the central mediator of tumor angiogenesis, stimulating the growth of new blood vessels from nearby capillaries and allowing tumors to access the oxygen and nutrients they need to grow [51]. 

**Figure 6:**
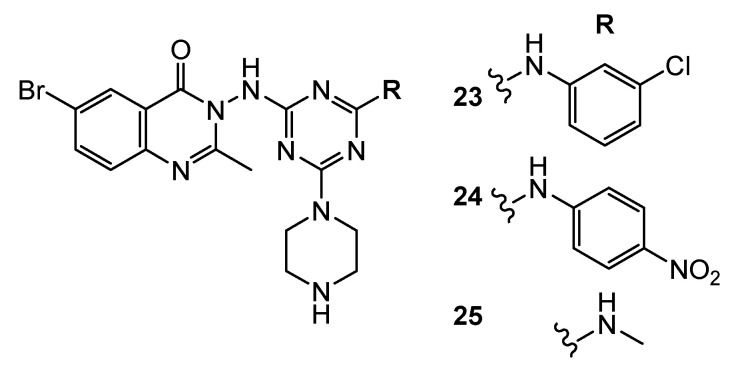
Structure of VEGF inhibitors.

Quinazoline-1,3,5-triazine derivatives **23**, **24,** and **25** (Figure 6) demonstrated antitumor activity against HeLa, MCF-7, HL-60, and HepG2 with IC_50_ values in range of 6–16 µM. In addition, they were non-toxic against the normal cell line of HFF (human foreskin fibroblasts). Molecular docking results demonstrated the high potency of derivatives **23**, **24** and **25** to bind the hydrophobic pocket of the N-terminal chain in the ATP binding site of VEGFR [52].

### 2.7. Focal Adhesion Kinase Inhibitors

Focal Adhesion Kinase (FAK) is a 125-kDa cytoplasmic tyrosine kinase. Deregulation of FAK-dependent processes such as cell adhesion, growth, survival, and mobility are a significant component of tumor progression. Overexpression of FAK leads to the inhibition of apoptosis and an increase in the incidence of metastatic tumors [53].

**Figure 7:**
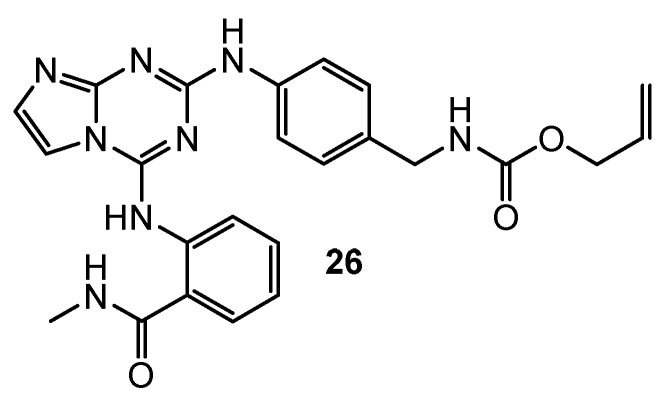
Structure of FAK inhibitor.

Dao et al. showed that compound **26** (Figure 7) is the strongest FAK inhibitor (IC_50_ = 0.05 µM). Growth inhibitory activity on human glioblastoma (U-87MG), human colon carcinoma (HCT-116), MDA-MB-231, and human prostate cancer (PC-3) by compound **26** obtained the following results 0.42 µM, 0.13 µM, 0.14 µM, and 0.63 µM compared to TAE-226 (0.19 µM, 0.23 µM, 1.9 µM, and 0.26 µM). Furthermore, compound **26** turned out to fit well into the ATP binding site of the FAK via molecular docking [16].

### 2.8. Ubiquitin Conjugating Enzyme Inhibitors

**Figure 8:**
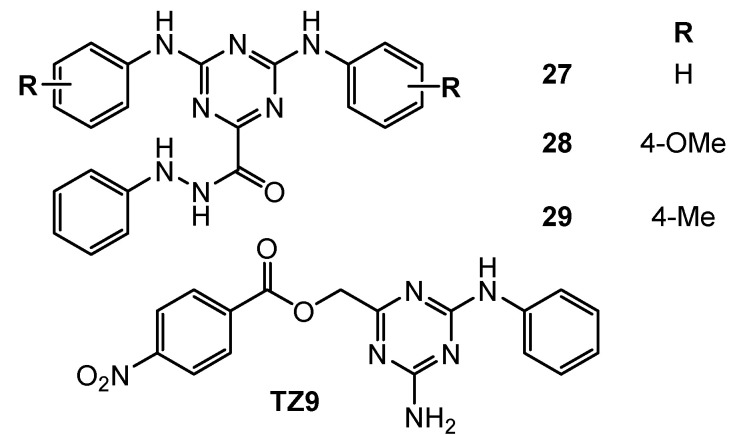
Structure of ubiquitin conjugating enzyme inhibitors.

RAD6, an E2 ubiquitin-conjugating enzyme, is overexpressed in many cancer cells and is responsible for the positive regulation of β-catenin, its stabilization and activity. N’-phenyl-4,6-bis(arylamino)-1,3,5-triazine-2-carbohydrazides derivatives **27**–**29** (Figure 8) were evaluated for their ability to inhibit Rad6B ubiquitin conjugation in the human cancer cell lines: OV90 (ovarian cancer), H1299 (human non-small cell lung carcinoma), A549, MCF-7, MDA-MB231, and HT-29 (colon cancer). For all of the examined compounds lower than for TZ9 IC_50_ values were obtained (3.3–22 µM) (Figure 8) [17].

### 2.9. Primary Anticancer Studies

**Figure 9:**
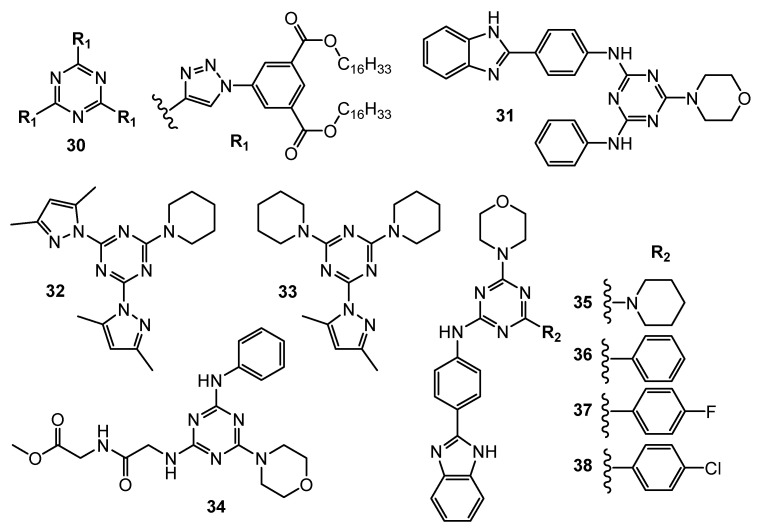
Structures of compounds **30–38**.

Compound **30** (Figure 9) obtained via the click chemistry method showed higher potency than doxorubicin. Derivative **30** exhibited an IC_50_ against MCF-7 and HepG2 cells of 2.95 µg/mL and 3.70 µg/mL, respectively, and showed no toxic activity against the growth of normal HFB4 cells [18]. 

Interesting results have emerged from the comparison of the antitumor properties of the two groups of 1,3,5-triazine derivatives. The groups differed only in one substituent, the first group contained chlorine and the second group contained morpholine. In the second case, a noticeable increase in cytotoxic activities was observed. According to cancer cell lines MCF-7, MDAMB-231, HT-29, HGC-27 the derivative **31** (Figure 9) proved to be most potent with IC_50_ values of 4.8 µM, 8.3 µM, 9.8 µM, and 15.1 µM [19].

Pyrazolyl-1,3,5-triazine derivatives were tested in vitro against MCF 7, MDA-MB-231, HepG2, LoVo (colorectal carcinoma) and K-562 (leukemia). Compounds **32** and **33** (Figure 9) demonstrated IC_50_ values within the range of 5 to 9 μM. An in vivo test on a zebrafish proved the non-toxicity of compounds **32** and **33** [20].

Trisubstituted s-triazine derivatives containing morpholine/piperidine, anilines, and dipeptides were evaluated for their anticancer activity against MCF-7 and MDA-MB-231. Among the 15 synthesized compounds, analog **34** (Figure 9) elicited the highest inhibitory properties against MCF-7 (IC_50_ = 0.82 µM). Moreover MCF-7 cells were significantly arrested in the G2/M stage. An in vivo studies of **34** in zebrafish presented non-toxic properties [21].

A novel series of triazine-benzimidazole analogs were synthesized and their antiproliferative activity against 60 human cancer cell lines was evaluated. Screening data revealed that triazine substituted with piperidine **35**, phenyl **36**, 4-fluorophenyl **37**, and 4-chlorophenyl **38** (Figure 9) presented the highest inhibiting potency [22]. 

**Figure 10:**
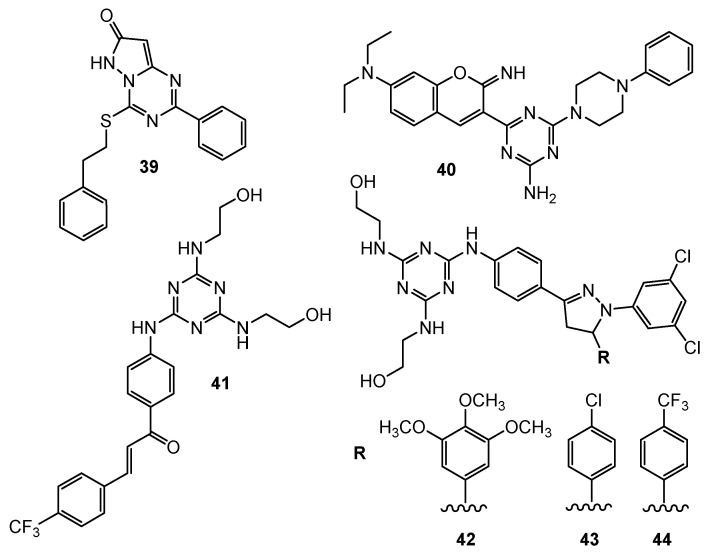
Structures of compounds **39**–**44**.

4-Phenethylthio-2-phenylpyrazolo[1,5-a][1,3,5]triazin-7(6H)-one **39** (Figure 10) was designed and synthesized as a potential anticancer agent. An in vitro evaluation of its antiproliferative activity against A549 and MDA-MB231 confirmed the assumption. The test results were not good enough. On the other hand, modifications of the obtained structure may contribute to the improvement of anti-cancer properties [23].

The series of novel hybrid molecules formed from 2,4-diamino-1,3,5-triazine and 2-iminocoumarin were tested toward the human pancreatic cancer cell line DAN-G, human A-427, human non-small cell lung cancer cell line LCLC-103H, human cervical cancer cell line SISO, and human urinary bladder cancer cell line RT-4. Compound **40** (Figure 10) presented the following values IC_50_: 2.14 µM, 1.51 µM, 2.21 µM, 2.60 µM, and 1.66 µM [24].

Moreno et al. designed and synthesized 28 1,3,5-triazine-based 2-pyrazolines. In vitro tests were conducted against 58 different human tumor cell lines. The first stage of research checked mean growth and growth inhibition, and identified four compounds **41**–**44** (Figure 10) with the lowest value (%). In the next step, the inhibitory activity of compounds **41**–**44** in terms of GI_50_ and LC_50_ was verified, determining the most susceptible carcinoma cell lines [25].

**Figure 11:**
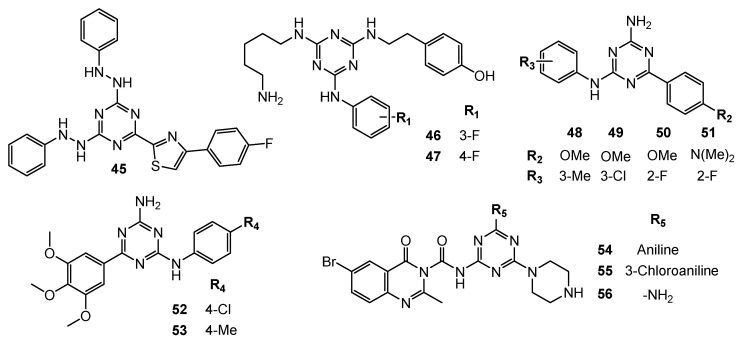
Structures of compounds **45**–**56**.

Wang et al. presented 16 compounds containing a phenylhydrazine and a thiazole moiety. Halogen-containing compound **45** (Figure 11) showed an uttermost inhibitory effect against MDA-MB-231, HeLa, KG1a (acute myelogenous leukaemia), and Jurkat (T-cell leukaemia) cancer cells. Subsequently cervical cancer cells (SiHa, CaSki, DoTc2) were treated with compound **45**, and the obtained IC_50_ values were in the range from 1.34 µg/mL to 4.56 µg/mL. An in vivo test on the nude mouse xenograft model revealed inhibition potency of compound **45** by the reduction of tumor volume [26].

The 2-(fluorophenylamino)-4,6-disubstituted 1,3,5-triazine induced inhibition of inflammation and cancer growth. SAR studies underlined the important role of 3- and 4-fluorphenylamino moiety **46** and **47** (Figure 11). Compound **47** significantly reduced tumor tissue in several animal models and decreased PC-3 proliferation with an IC_50_ value of 20 µM. This analog also arrested PC-3 cells in stage G0/G1 [27].

Via three-components one spot condensation 110 new of 1,3,5-triazine derivatives were obtained. Antiproliferative activity of the most potent compounds **48**–**51** (Figure 11) identified in the screening against DU145 prostate-cancer cells had GI_50_ values of 3.43 µM, 4.01 µM, 2.38 µM and 0.67 µM, respectively [28]. Subsequent studies generated further derivatives that were tested for three breast tumors. Evaluation led to the determination that the most active structures are **52** and **53** (Figure 11) and indicated that the group of derivatives were more active against triple negative breast cancer MDA-MB231 [29,30].

Derivatives based on quinazoline combined with a 1,3,5-triazine ring via urea bridge presented antitumor activity against TPC-1 cells (thyroid cancer), MCF-7. Corresponding to the normal cell line (human foreskin fibroblasts), compounds **54**–**56** (Figure 11) were non-toxic. In addition, these structures showed the best IC_50_ values against carcinoma cells, and demonstrated tyrosine kinase inhibitory potency [31].

**Figure 12:**
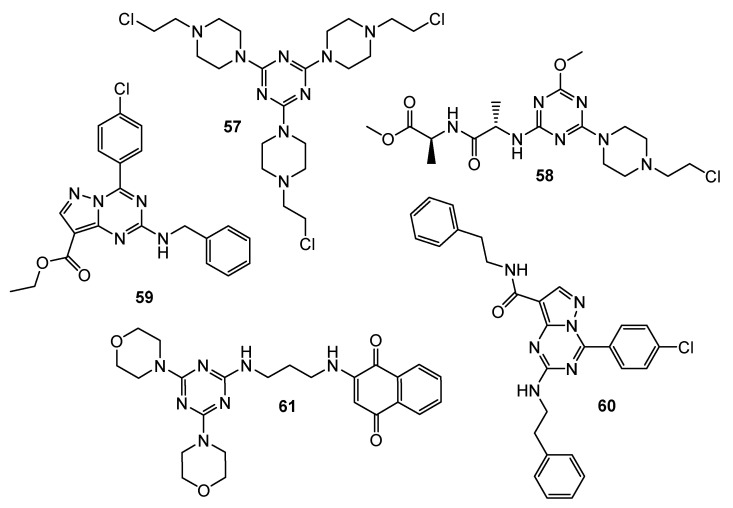
Structures of compounds **57**–**61**.

Mono-, di- and tri-2-chloroethylamine-1,3,5-triazine derivatives were confronted with glioblastoma cells. An in vitro study detailed trisubstituted triazine **57** (Figure 12) was the most relevant cytotoxic molecule with IC50 values equal 46 μM, 50 μM, and 40 μM for LBC3, LN-18 and LN-229 cell lines, respectively [32]. Anticancer activity of mono-2-chloroethylamine-1,3,5-triazine derivatives bearing dipeptide were proven on DLD-1 and HT-29 cell lines. The most perspective structure was **58** (Figure 12), which presented IC_50_ values of 13.71 μM and 17.78 μM, for DLD-1 and HT-29, respectively. 5-fluorouracil exhibited lower activity as a reference. Compound **58** increased the expression of BAX and decreased the amount of Bcl-2 both in DLD-1 and in HT-29 [33].

A total of thirty-four novel pyrazolo[1,5-a][1,3,5]triazine derivatives were screened against 60 cancer cell lines. Results suggested that the most antiproliferative compounds were **59** and **60** (Figure 12). Analog **59** exhibited% inhibition ranging from 40% to 115%, and 82.38% for CDK2, and derivative **60** exhibited% inhibition ranging from 43% to 92%, and 81.96% for CDK2 [34].

Hybrid molecule containing 1,4-naphthoquinone, 1,3,5-triazine and morpholine **61** (Figure 12) turned out to be strongly complexed with PI3Kγ and AMPK (5′ AMP-activated protein kinase) during docking studies. Analog **61** had an IC_50_ value of approximately 25 μM when exposed to the SKMEL-103 (N-RAS mutated) cell line. A Western blot determined the decreased expression of both PI3Kγ and AMPK [35].

**Figure 13:**
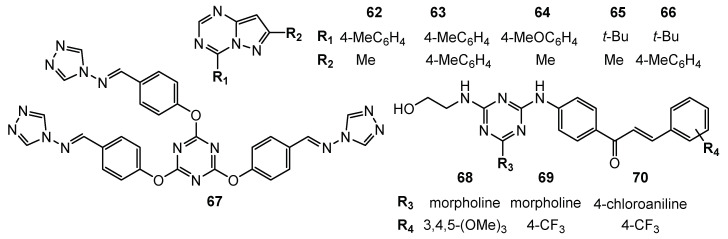
Structures of compounds **61**–**70**.

Screening studies of 2-(dichloromethyl)pyrazolo[1,5-a][1,3,5]triazines **62**–**66** (Figure 13) showed potential anticancer properties against non-small cell lung cancers, colon cancers, renal cancer, etc. [36].

Prepared 4-amino-1,2,4-triazole Schiff base derivative **67** (Figure 13) was verified as an antitumor agent. The IC_50_ value of **67** was equal to 144.1 µg/mL for A549 and 195.6 µg/mL for the human hepatoma cell line (Bel7402) [37].

From the series of novel chalcone- and pyrazoline-based 1,3,5-triazines derivatives, compounds **68**–**70** (Figure 13) demonstrated the best potent in vitro anticancer activity with GI50 values significantly lower than reference drug 5-FU. Chalcone **68** showed GI_50_ values in the range of 0.422–3.05 µM, with the SR cell line (leukemia, GI50 = 0.422 µM) being the most sensitive strain. Compound **69** exhibited GI50 values in the range of 1.25–8.66 µM, with the MCF7 (GI50 = 1.25 µM) being the most sensitive strain, while compound **70** showed GI50 values in the range of 1.48–14.9 µM, being especially effective against HCT-116 with GI50 = 1.48 µM. The best cytotoxicity value was shown by compound **69** against UO-31 (renal cancer, LC50 = 5.08 µM) [38].

## 3. Search Strategy and Selection Criteria

The aim of this study was to collect knowledge and data on the synthesized novel 1,3,5-triazine derivatives, their effects on cancer cells, and to identify enzymes as potential targets for these substances. To carry out the study, the following databases were searched: PubMed (NCBI), Web of Science, and Scopus, using the following key words: 1,3,5-triazine, s-triazine, anticancer, antitumor, and enzyme inhibitor. We examined original articles and case studies published between 2015 and 2021. The results of the study include the compounds from papers with the highest activity.

## 4. Conclusions

The “hybrid” approach incorporating a triazine framework ensures an improved profile against the target biological pathways pertaining to infectious parasites, microbes, and conditions such as cancer and neurodegeneration. The multi-targeting approach of the hybrid compounds ensures an effective overcoming of the key regulatory pathways contributing to complicacies such as drug resistance. This review presents a comprehensive discussion on the candidature of the 1,3,5-triazine scaffold for a rational development of the hybrid molecules by conjugation with bioactive pharmacophoric moieties. The basis of superior efficacy of 1,3,5-triazine based hybrid molecules by considering their interactions with the cellular targets has also been discussed in a succinct manner. The literature revealed that s-triazine derivatives possess diverse anticancer potential, easy synthetic routes for synthesis, and have attracted researchers for development of new chemotherapeutic agents. Extensive research is required on the 1,3,5-triazine moiety to find novel analogs suitable for clinical applications in cancer treatment.

## Figures and Tables

**Table 1:** Promising effects of 1,3,5-triazine derivatives on cell lines and/or enzymes. N/A; not available.

No.	Cancer Cells/Effects	Targets/Effects	Reference Substance	Ref.
**1**	N/A	DNA topoisomerase IIα (IC50 = 57.6 µM)	Etoposide: DNA topoisomerase IIα (IC_50_ = 59.2 µM)	[3]
**2**	A549 (IC_50_ = 0.20 µM) MCF-7 (IC_50_ = 1.25 µM) Hela (IC_50_ = 1.03 µM)	PI3Kα (IC_50_ = 7.0 nM) mTOR (IC_50_ = 48 nM)	GDC-0941: A549 (IC50 = 1.21 µM), MCF-7 (IC50 = 1.47 µM), Hela (IC50 = 3.72 µM), PI3Kα (IC_50_ = 6.0 nM), mTOR (IC_50_ = 525 nM); PI-103: PI3Kα (IC_50_ = 5.1 nM), mTOR (IC_50_ = 21 nM)	[4]
**3**	MDA-MB321 (IC50 = 15.83 µM) MCF-7 (IC50 = 16.32 µM) Hela (IC50 = 2.21 µM) HepG2 (IC50 = 12.21 µM)	mTOR (IC50 = 8.45 nM) PI3Kα (IC50 = 3.41 nM)	Gedatolisib: mTOR (IC_50_ = 2.5 nM) PI3Kα (IC_50_ = 6.04 nM)	[5]
**7**	leukemia (GI50 = 1.96 µM) colon cancer (GI50 = 2.60 µM) CNS (GI50 = 2.72 µM) melanoma (GI50 = 1.91 µM) ovarian (GI50 = 4.01 µM) renal (GI50 = 3.03 µM) prostate (GI50 = 4.40 µM) breast (GI50 = 2.04 µM)	hDHFR (IC50 = 0.002 µM)	Triazine–Benzimidazole: leukemia (GI_50_ = 3.71 µM) colon cancer (GI_50_ = 2.76 µM) CNS (GI_50_ = 1.86 µM) melanoma (GI_50_ = 2.70 µM) ovarian (GI_50_ = 2.41 µM) renal (GI_50_ = 1.89 µM) prostate (GI_50_ = 2.75 µM) breast (GI_50_ = 2.58 µM) MTX: hDHFR (IC_50_ = 0.02 µM)	[6]
**8**	HCT116 (IC50 = 0.88 µM) A549 (IC_50_ = 0.07 µM) HL-60 (IC50 = 0.33 µM)	hDHFR (IC_50_ = 0.00746 µM)	MTX: HCT116 (IC_50_ = 0.75 µM) A549 (IC_50_ = 0.25 µM) HL-60 (IC_50_ = 1.09 µM) HepG2 (IC_50_ = 0.41 µM) MDA-MB-234 (IC_50_ = 9.49 µM) hDHFR (IC50 = 0.00667 µM)	[7]
**9**	HCT116 (IC50 = 1.61 µM) A549 (IC50 = 0.5 µM) HL-60 (IC50 = 0.87 µM)	hDHFR (IC50 = 0.00372 µM)		
**10**	HCT116 (IC50 = 0.02 µM) A549 (IC50 = 0.74 µM) HL-60 (IC50 = 0.35 µM) HepG2 (IC50 = 1.4 µM) MDA-MB-234 (IC50 = 0.44 µM)	hDHFR (IC50 = 0.00646 µM)		
**11**	HCT116 (IC50 = 0.001 µM) A549 (IC50 = 0.21 µM) HL-60 (IC50 = 0.33 µM) HepG2 (IC50 = 1.38 µM) MDA-MB-234 (IC50 = 0.06 µM)	hDHFR (IC50 = 0.00408 µM)		
**12**	HCT116 (GI50 = 0.026 µM) MCF-7 (GI50 = 0.08 µM)	hDHFR (IC50 = 0.0061 µM) rat TrxR (IC50 = 4.6 µM)	MTX: hDHFR (IC_50_ = 0.0079 µM) HCT116 (GI_50_ = 0.015 µM) MCF-8 (GI_50_ = 0.024 µM)	[8]
**13**	HCT116 (GI50 = 0.116 µM) MCF-8 (GI50 = 0.127 µM)	hDHFR (IC50 = 0.0026 µM) rat TrxR (IC50 = 5.9 µM)		
**14**	HeLa (IC50 = 16 µM) HaCaT (IC50 = 61 µM)	hCAI (K_I_ = 733.3 nM) hCAII (K_I_ = 160.8 nM) hCAIX (K_I_ = 41.1 nM) hCAXII (K_I_ = 77.6 nM)	AAZ: hCAI (KI = 250 nM) hCAII (KI = 12.1 nM) hCAIX (KI = 25.8 nM) hCAXII (KI = 5.7 nM) MZA: hCAI (K_I_ = 780 nM) hCAII (K_I_ = 14 nM) hCAIX (K_I_ = 27 nM) hCAXII (K_I_ = 3.4 nM) EZA: hCAI (K_I_ = 25 nM) hCAII (K_I_ = 8 nM) hCAIX (K_I_ = 34 nM) hCAXII (K_I_ = 22 nM) DCP: hCAI (K_I_ = 1200 nM) hCAII (K_I_ = 38 nM) hCAIX (K_I_ = 50 nM) hCAXII (K_I_ = 50 nM)	[9]
**15**	N/A	hCAI (KI = 16.7 nM) hCAII (KI = 7.4 nM) hCAIX (KI = 0.4 nM)		[10]
**16**	N/A	hCAI (KI = 2679.1 nM) hCAII (KI = 380.5 nM) hCAIX (KI = 27.0 nM)		
**17**	N/A	hCAI (KI = 394.9 nM) hCAII (KI = 3.1 nM) hCAIX (KI = 0.91 nM)		[11]
**18**	N/A	hCAI (KI = 441.7 nM) hCAII (KI = 152.9 nM) hCAIX (K = 14.6 nM) hCAXII (KI = 44.4 nM)		[12]
**19**	HeLa (IC50 = 39.7 µM) MCF-7 (IC50 = 41.5 µM) HL-60 (IC50 = 23.1 µM) HepG2 (IC50 = 31.2 µM)	EGFR-TK (Inhibition rate = 94.3%; C = 10 µM)	Cisplatin: HeLa (IC_50_ = 32.5 µM) MCF-7 (IC_50_ = 24.4 µM) HL-60 (IC_50_ = 12.3 µM) HepG2 (IC_50_ = 25.9 µM) Erlotinib: EGFR-TK (Inhibition rate = 100%; C = 10 µM);	[13]
**20**	N/A	EGFR-TK (IC50 = 2.54 µM)	Dacomitinib: EGFR-TK (IC_50_ = 0.06 µM)	[14]
**21**	HeLa (IC50 = 44.5 µM) MCF-7 (IC50 = 52.2 µM) HL-60 (IC50 = 40.3 µM) HepG2 (IC50 = 56.4 µM)	EGFR-TK (Inhibition rate = 96.3%; C = 10 µM)	Cisplatin: HeLa (IC_50_ = 31.3 µM) MCF-7 (IC_50_ = 22.5 µM) HL-60 (IC_50_ = 14.3 µM) HepG2 (IC_50_ = 26.4 µM) Erlotinib: EGFR-TK (Inhibition rate = 100%; C = 10 µM)	[15]
**22**	HeLa (IC50 = 32.4 µM) MCF-7 (IC50 = 32.3 µM) HL-60 (IC50 = 26.3 µM) HepG2 (IC50 = 45.3 µM)	EGFR-TK (Inhibition rate = 90.5%; C = 10 µM)		
**26**	U-87MG (IC50 = 0.42 µM) HCT-116 (IC50 = 0.13 µM) MDA-MB-231 (IC50 = 0.14 µM) PC-3 (IC50 = 0.63 µM)	FAK (IC_50_ = 50 nM)	TAE-226: U-87MG (IC_50_ = 0.19 µM) HCT-116 (IC_50_ = 0.23 µM) MDA-MB-231 (IC_50_ = 1.9 µM) PC-3 (IC_50_ = 0.26 µM) FAK (IC50 = 7 nM)	[16]
**27**	HT-29 (IC50 = 9.5 µM) H1299 (IC50 = 11 µM) A549 (IC50 = 14.6 µM) MDA-MB-231 (IC50 = 2.5 µM) OV90 (IC50 = 8 µM) A2780 (IC50 = 7.1 µM) MCF-7 (IC50 = 6 µM)	Rad6 ubiquitin conjugating enzyme (nd)	TZ9: HT-29 (IC_50_ = 8.3 µM) H1299 (IC_50_ = 45 µM) A549 (IC_50_ = 7.2 µM) MDA-MB-231 (IC_50_ = 4.6 µM) OV90 (IC_50_ = 60 µM) A2780 (IC_50_ = 7.8 µM) MCF-7 (IC_50_ = 5 µM)	[17]
**28**	HT-29 (IC50 = 5.8 µM) H1299 (IC50 = 5 µM) A549 (IC50 = 10.8 µM) MDA-MB-231 (IC50 = 4.2 µM) OV90 (IC50 = 12 µM) A2750 (IC50 = 6.3 µM) MCF-7 (IC50 = 7.2 µM)			
**29**	HT-29 (IC50 = 5.2 µM) H1299 (IC50 = 22 µM) A549 (IC50 = 11.6 µM) MDA-MB-231 (IC50 = 3.5 µM) OV90 (IC50 = 5 µM) A2750 (IC50 = 3.6 µM) MCF-7 (IC50 = 4.2 µM)			
**30**	MCF-7 (IC50 = 2.95 µg/mL) HepG2 (IC_50_ = 3.7 µg/mL)	N/A	Doxorubicin: MCF-7 (IC_50_ = 2.98 µg/mL) HepG2 (IC_50_ = 3.82 µg/mL)	[18]
**31**	MCF-7 (IC50 = 4.8 µM) MDA-MB-231 (IC50 = 48.3 µM) HT-29 (IC50 = 9.8 µM) HGC-27 (IC50 = 15.1 µM)	N/A	ZSTK474: MDA-MB-231 (IC_50_ = 10.8 µM) HT-29 (IC_50_ = 25.1 µM) HGC-27 (IC_50_ = 1.11 µM)	[19]
**32**	MCF7 (IC50 = 5 µM) MDA-MB-231 (IC50 = 15 µM) HepG2 (IC50 = 21.1 µM) LoVo (IC50 = 8.4 µM) K-562 (IC50 = 5.9 µM)	Arrest cell proliferation in S and G2/M phase. None lethal for zebrafish embryos.	N/A	[20]
**33**	MCF7 (IC50 = 7.5 µM) MDA-MB-231 (IC50 = 14 µM) HepG2 (IC50 = 17.5 µM) LoVo (IC50 = 6.1 µM) K-562 (IC50 = 9.8 µM)			
**34**	MCF-7 (IC50 = 0.82 µM) MDA-MB-231 (IC50 = 9.36 µM) HCT-116 (IC50 = 17.89 µM)	Arrest of MCF-7 cells in the G2/M stage(36.8%). Mortality response of zebrafish embryos—na.	Tamoxifen: MCF-7 (IC_50_ = 5.12 µM) MDA-MB-231 (IC_50_ = 15.01 µM) HCT-116 (IC_50_ = 26.41 µM)	[21]
**35**	MG-MID (GI50 = 2.68 µM; TGI = 11 µM; LC50 = 32.3 µM)	BSA (distance in complex = 7.9 nm)	N/A	[22]
**36**	MG-MID (GI50 = 1.38 µM; TGI = 3.15 µM; LC50 = 8.63 µM)	BSA (distance in complex = 6.61 nm)		
**37**	MG-MID (GI50 = 2.37 µM; TGI = 7.16 µM; LC50 = 7.88 µM)	BSA (distance in complex = 7.62 nm)		
**38**	MG-MID (GI50 = 0.72 µM; TGI = 1.8 µM; LC50 = 4.88 µM)	BSA (distance in complex = 7.98 nm)		
**39**	A549 (IC50 = 53 µM)	N/A	Floxuridine: A549 (IC_50_ = 5.8 µM)	[23]
**40**	DAN-G (IC50 = 2.14 µM) A-427 (IC50 = 1.51 µM) LCLC-103H (IC50 = 2.21 µM) SISO (IC50 = 2.6 µM) RT-4 (IC50 = 1.66 µM)	Ct-DNA (potencial target)	Cisplatin: DAN-G (IC_50_ = 0.73 µM)A-427 (IC_50_ = 1.96 µM) LCLC-103H (IC_50_ = 0.90 µM) SISO (IC_50_ = 0.24 µM) RT-4 (IC_50_ = 1.61 µM)	[24]
**41**	UO-31 (GI50 = 1.54 µM)	N/A	N/A	[25]
**42**	RXF 393 (GI50 = 0.569 µM) HS 578 (GI50 = 0.644 µM)			
**43**	SF-539 (GI50 = 1.35 µM)			
**44**	SF-539 (GI50 = 1.18 µM)			
**45**	MDA-MB-231 (IC50 = 4.3 µg/mL) HeLa (IC50 = 2.21 µg/mL) KG1a (IC50 = 6.45 µg/mL) Jurkat (IC50 = 28.33 µg/mL) SiHa (IC50 = 1.34 µg/mL) CaSki (IC50 = 4.56 µg/mL) DoTc2 (IC50 = 2.15 µg/mL)	Increase concentration of C-caspase-3, C-caspase-9 and Bcl-2. Decrease of Bax. Tumor reduction in nude mouse (C = 10 µM).	Erlotinib: MDA-MB-231 (IC_50_ = 0.16 µg/mL) HeLa (IC_50_ = 0.21 µg/mL) KG1a (IC_50_ = 0.18 µg/mL) Jurkat (IC_50_ = 22.43 µg/mL) SiHa (IC_50_ = 0.25 µg/mL) CaSki (IC_50_ = 0.34 µg/mL) DoTc2 (IC_50_ = 0.28 µg/mL)	[26]
**46**	N/A	TNF-α (IC_50_ = 29 µM)	N/A	[27]
**47**	PC-3 (IC50 = 43.3 µM)	TNF-α (IC_50_ = 13 µM), inducing cell-cycle arrest at the G0/G1 phase (J774 cell line).		
**48**	DU145 (GI50 = 3.43 µM)	N/A	Nilotinib: DU145 (GI_50_ = 6.35 µM)	[28]
**49**	DU145 (GI50 = 4.01 µM)			
**50**	DU145 (GI50 = 2.38 µM)			
**51**	DU145 (GI50 = 0.67 µM)			
**52**	MDA-MB231 (GI_50_ = 0.007 µM) SKBR-3 (GI_50_ = 0.3 µM) MCF-7 (GI_50_ = 12.5 µM)	N/A	MTX: MDA-MB231 (GI50 = 0.01 µM) MCF-7 (GI50 = 5.79 µM) Nilotinib: MDA-MB231 (GI_50_ = 0.04 µM) SKBR-3 (GI_50_ = 9.6 µM)	[29,30]
**53**	MDA-MB231 (GI50 = 0.001 µM) SKBR-3 (GI50 = 0.21 µM)			
**54**	MCF-7 (IC50 = 14.85 µM) TPC-1 (IC50 = 9.23 µM)	Phosphorylated TK (Inhibition rate = 94.4%; C = 10 µM)	Vandatinib: MCF-7 (IC_50_ = 10.42 µM) TPC-1 (IC_50_ = 7.63 µM) Phosphorylated TK (Inhibition rate = 98.6%; C = 10 µM)	[31]
**55**	MCF-7 (IC50 = 12.5 µM) TPC-1 (IC50 = 7.16 µM)	Phosphorylated TK (Inhibition rate = 96.4%; C = 10 µM)		
**56**	MCF-7 (IC50 = 14.43 µM) TPC-1 (IC50 = 8.8 µM)	Phosphorylated TK (Inhibition rate = 94.3%; C = 10 µM)		
**57**	LN-18 (IC50 = 46 µM) LN-229 (IC50 = 50 µM) LBC3 (IC50 = 40 µM)	N/A	N/A	[32]
**58**	DLD-1 (IC50 = 13.71 µM) HT-29 (IC50 = 17.78 µM)	BAX (increase); Bcl-2 (decrease)	5-FU: DLD-1 (IC_50_ = 27.22 µM) HT-29 (IC_50_ = 21.72 µM)	[33]
**59**	HCT-116 (Inhibition = 115.53%) SW-620 (Inhibition = 95.06%) SF-539 (Inhibition = 89.27%) OVCAR-4 (Inhibition = 94.39%) PC786-0 (Inhibition = 93.76%) ACHN (Inhibition = 86.27%) MCF-7 (Inhibition = 94.82%)	CDK2 (Inhibition rate = 82.38%; C = 10 µM; IC50 = 1.85 µM)	Roscovitine: CDK2 (Inhibition rate = 89.6%; C = 10 µM)	[34]
**60**	ATCC (Inhibition = 90.02%) NCI-H460 (Inhibition = 83.66%) OVCAR-4 (Inhibition = 92.27%)	CDK2 (Inhibition rate = 81.96%; C = 10 µM; IC50 = 2.09 µM)		
**61**	SKMEL-103 (IC50 = 25 µM)	PI3K (decrease)AMPK (decrease)	N/A	[35]
**62**	NCI-H460 (Growth Percent = −50%) MDA-MB468 (Growth Percent = −20.7%)	N/A	N/A	[36]
**63**	HCC-2998 (Growth Percent = −82.1%) RXF 393 (Growth Percent = −68%) NCI-H460 (Growth Percent = −58.3%) ACHN (Growth Percent = −57%) MDA-MB-468 (Growth Percent = −52.3%)			
**64**	HCC-2998 (Growth Percent = −69.3%) RXF 393 (Growth Percent = −66%) NCI-H460 (Growth Percent = −64.8%) ACHN (Growth Percent = −45%)			
**65**	HCC-2998 (Growth Percent = −77%) RXF 393 (Growth Percent = −74.4%) NCI-H460 (Growth Percent = −49.4%) MDA-MB-468 (Growth Percent = −47%)			
**66**	HCC-2998 (Growth Percent = −53.7%) RXF 393 (Growth Percent = −55%) NCI-H460 (Growth Percent = −54.7%) ACHN (Growth Percent = −52.8%) NCI-H322M (Growth Percent = −50.5%)			
**67**	A549 (IC50 = 144.1 µg/mL) Bel7402 (IC50 = 195.6 µg/mL)	N/A	N/A	[37]
**68**	leukemia (Mean GI50 = 0.96 µM) colon cancer (Mean GI50 = 1.64 µM) CNS (Mean GI50 = 1.80 µM) melanoma (Mean GI50 = 1.62 µM) ovarian (Mean GI50 = 2.12 µM) renal (Mean GI50 = 1.66 µM) prostate (Mean GI50 = 1.75 µM) breast (Mean GI50 = 1.59 µM)	N/A	N/A	[38]
**69**	leukemia (Mean GI50 = 2.55 µM) colon cancer (Mean GI50 = 1.92 µM) CNS (Mean GI50 = 2.09 µM) melanoma (Mean GI50 = 3.4 µM) ovarian (Mean GI50 = 2.67 µM) renal (Mean GI50 = 1.80 µM) prostate (Mean GI50 = 1.2.22 µM) breast (Mean GI50 = 2.03 µM)			
**70**	leukemia (Mean GI50 = = 4.14 µM) colon cancer (Mean GI50 = 1.92 µM) CNS (Mean GI50 = 3.13 µM) melanoma (Mean GI50 = 7.84 µM) ovarian (Mean GI50 = 6.05 µM) renal (Mean GI50 = 3.28 µM) prostate (Mean GI50 = 4.54 µM) breast (Mean GI50 = 3.42 µM)			

## Data Availability

Not applicable.
